# The role of medial prefrontal cortex in the working memory maintenance of one’s own emotional responses

**DOI:** 10.1038/s41598-018-21896-8

**Published:** 2018-02-22

**Authors:** Ryan Smith, Richard D. Lane, Anna Alkozei, Jennifer Bao, Courtney Smith, Anna Sanova, Matthew Nettles, William D. S. Killgore

**Affiliations:** Department of Psychiatry, University of Arizona, 1501 N. Campbell Ave, Tucson, AZ 85724-5002 United States

## Abstract

The role of medial prefrontal cortex (MPFC) in maintaining emotional information within working memory (WM) remains insufficiently investigated – with some studies suggesting this process activates MPFC and others suggesting its activity is suppressed. To reconcile these different results, we asked 26 healthy participants to complete a WM task involving the maintenance of emotional content (EWM), visual content (VWM), or no content (“rest”) after exposure to emotion-provoking images. We also assessed individual differences in emotional awareness (EA). We observed that dorsal MPFC was more active during EWM than VWM; further, relative to the rest condition, both of these WM conditions involved suppression of ventral MPFC. We also observed that the dorsal anterior cingulate subregion of dorsal MPFC was positively associated with EA. We discuss how these results may be able to reconcile the findings of previous EWM studies, and extend understanding of the relationship between MPFC, EA, and WM.

## Introduction

Working memory (WM) refers to the temporary maintenance and manipulation of information for use in guiding goal-directed decision-making and action selection^1–3^. Previous investigations of the neural basis of WM suggest this process involves interactions between executive control network (ECN) regions (e.g., lateral frontal-parietal regions) and other cortical regions that represent the information being maintained^3–9^. It is also widely recognized that these neural mechanisms overlap considerably with those of top-down attentional control. Specifically, based on an individual’s current goals, the ECN is thought to send top-down modulatory signals that (1) attentionally amplify goal-relevant stimulus representations and (2) suppress goal-irrelevant representations; after stimulus removal, these same top-down modulatory signals can also maintain goal-relevant representations in an active state (i.e., in WM) for use in guiding decision making^10–12^. To date, however, the investigation of such mechanisms has mainly focused on WM for visual and auditory information. As such, the processes involved in maintaining/manipulating other types of information have not been fully characterized.

One important example is the process of maintaining and manipulating information about emotions. While several studies have shown that top-down attention to one’s own emotions amplifies activation within the medial prefrontal cortex (MPFC) and other regions of the default mode network (DMN)(i.e., relative to a top-down visual attention condition;^13–19^), very few studies to date have examined emotional WM (EWM). Of those that have, a major focus has been on the emotions of others. For example, one previous study examined the process of maintaining the emotions perceived in the faces of others within an N-Back task (using basic emotion concepts; e.g., “angry,” “happy,” etc.), and showed that this process recruits ECN regions similar to those found in the studies of visual/auditory WM described above^20^. This study also observed related *reductions* in (i.e., suppression of) activation within the MPFC and other DMN regions during this process. These findings suggest that WM for emotional information may draw on the same ECN system as WM for other kinds of information. However, it was also possible that participants used an alternative auditory strategy to keep the emotional information in mind in this study (i.e., internally repeating an emotion word, instead of actually holding a conceptualized emotional feeling in mind). Thus, the correct interpretation of these results was unclear.

In a more recent study, we sought to clarify this using an experimental paradigm that also used images of emotional faces, but that allowed the direct contrast of WM for emotional feelings, images, and words^21^. In that study, we confirmed that ECN regions are activated during EWM, even when controlling for the possible use of alternative visual or auditory strategies; further, no DMN activation was observed. Collectively the evidence from these two studies therefore supports the idea that the ECN plays an important role in EWM. However, as both of these studies focused on the emotions of other people, it remains to be determined whether this pattern would also be seen when holding one’s own emotions in WM. Based on the amplification of MPFC/DMN activation previously observed during top-down attention to one’s own emotions mentioned above (e.g. ref.^17^), one might predict greater MPFC involvement for self-focused EWM (i.e., in addition to ECN involvement). In fact, one study has found preliminary support for this possibility^22^. In that study, participants showed increased activation within the MPFC (as well as ECN regions) when asked to hold the intensity of their own affective response in mind^22^. Thus, it is possible that MPFC plays a greater role in EWM for the self than it does in EWM for others, perhaps by constructing representations of self-related information about emotions – which are then amplified/maintained by the ECN during self-focused attention and EWM (i.e., as suggested by recent neural models of emotion processing^23–25^, and as supported by previous work linking MPFC to self-representation and self-reflection processes more generally; e.g., see refs^26–28^). However, as Waugh *et al*.’s study^22^ asked participant to hold the intensity of their emotions in mind, rather than emotion concepts (e.g., the concept of “anger”; as done in the other EWM studies described above), this finding would need to be replicated using emotion concepts to more fully support this hypothesis.

Further, although not previously examined, the trait variable of “emotional awareness” (EA) could also have important influences on MPFC activation during EWM. EA measures the degree to which one has learned to conceptualize affective responses in fine-grained ways^29^. For example, someone with low EA may only categorize affective responses in somatic terms (e.g., “sick” or “achy”) or in non-specific affective terms (e.g., “bad” or “good”). In contrast, someone with high EA is more likely to conceptualize affective responses in more granular terms (e.g., “sad” or “angry”). These more complex conceptualization processes are known to engage the MPFC/DMN^12,30,31^, and EA has previously been shown to predict differences in MPFC activation (i.e., specifically the dorsal anterior cingulate [dACC] subregion;^32,33^). It is therefore plausible to hypothesize that EA may moderate MPFC activation during EWM. This is also consistent with our previous finding that EA was positively correlated with EWM performance with regard to the emotions of others^21^. Further, higher EA has previously been shown to predict better outcomes during psychotherapy^34^ – a context in which individuals are specifically asked to maintain information about their own emotions in mind in order to better understand them and find more adaptive responses to them. Combined with other related work (i.e., reviewed in refs^35–37^), this highlights the potential for important and clinically relevant interactions between EA and self-focused EWM processes.

In summary, while there is evidence to suggest that WM for the emotions of others recruits the ECN, and that it inhibits the MPFC/DMN (similar to WM for visual/auditory information), it is currently unclear whether this is also true of WM for one’s own emotions. Studies of attention to one’s own emotions (e.g. ref.^17^), and WM for the intensity of one’s own emotions^22^, both suggest the MPFC/DMN activation may instead be amplified by the ECN during self-focused EWM; further, MPFC has been implicated in other self-referential processes as well^27^. However, to date the only study that has investigated the neural basis of WM for one’s own emotions focused on emotion intensity rather than on emotion concepts, which does not allow a clear comparison^22^.

In the present study, our primary aim was to further examine the neural basis of WM for one’s own emotions. To do so, we modified a widely used task for studying attention to one’s own emotions, which presents participants with normatively pleasant, unpleasant, and emotionally neutral images^13–19^; this task is known to activate MPFC during emotion-focused attention (i.e., relative to exteroceptive [visual] attention). We modified this task to include (1) a delay period requiring maintenance of information in WM, and (2) the use of basic emotion concept categories. These modifications allowed us to directly test the hypothesis that holding one’s own conceptualized affective responses in WM would amplify, as opposed to inhibit, MPFC activation (i.e., unlike EWM for other people’s emotions, and similar to both top-down attention to one’s own emotions and MPFC activation observed in other self-directed cognitive processes). This hypothesis also follows from previously published neural models of emotion processing^23–25^, which suggest that top-down modulatory signals from the ECN function to amplify/maintain self-related emotion concept representations in the DMN when they are goal-relevant. As a secondary aim of the study, we also examined the association between two performance measures of EA in relation to MPFC/dACC activity during this task. This was done because WM for one’s own emotions plausibly requires awareness of those emotions, and also because we wished to assess whether MPFC/dACC activity during emotion-focused WM was influenced by EA in a similar manner to that observed in previous studies of emotion-focused attention^32,33^. We therefore hypothesized that MPFC/dACC activation during WM for emotions would be positively related to EA.

## Methods

### Participants

We recruited twenty-six adults (13 female; mean age = 23.12 ± 4.03) from the general population (using flyers and internet advertisements) to participate in the current study. Exclusion criteria included any history of psychiatric or neurological disorders (assessed via a phone screen questionnaire based on criteria within the Diagnostic and Statistical Manual for Mental Disorders, 4^th^ edition; DSM-IV-TR). All participants provided written informed consent prior to engaging in any study-related activities. All participants also received financial compensation for participation. The Institutional Review Board of the University of Arizona reviewed and approved the research protocol of the present study, and all methods were carried out in accordance with the relevant guidelines and regulations.

### Working Memory Task

**Figure 1:**
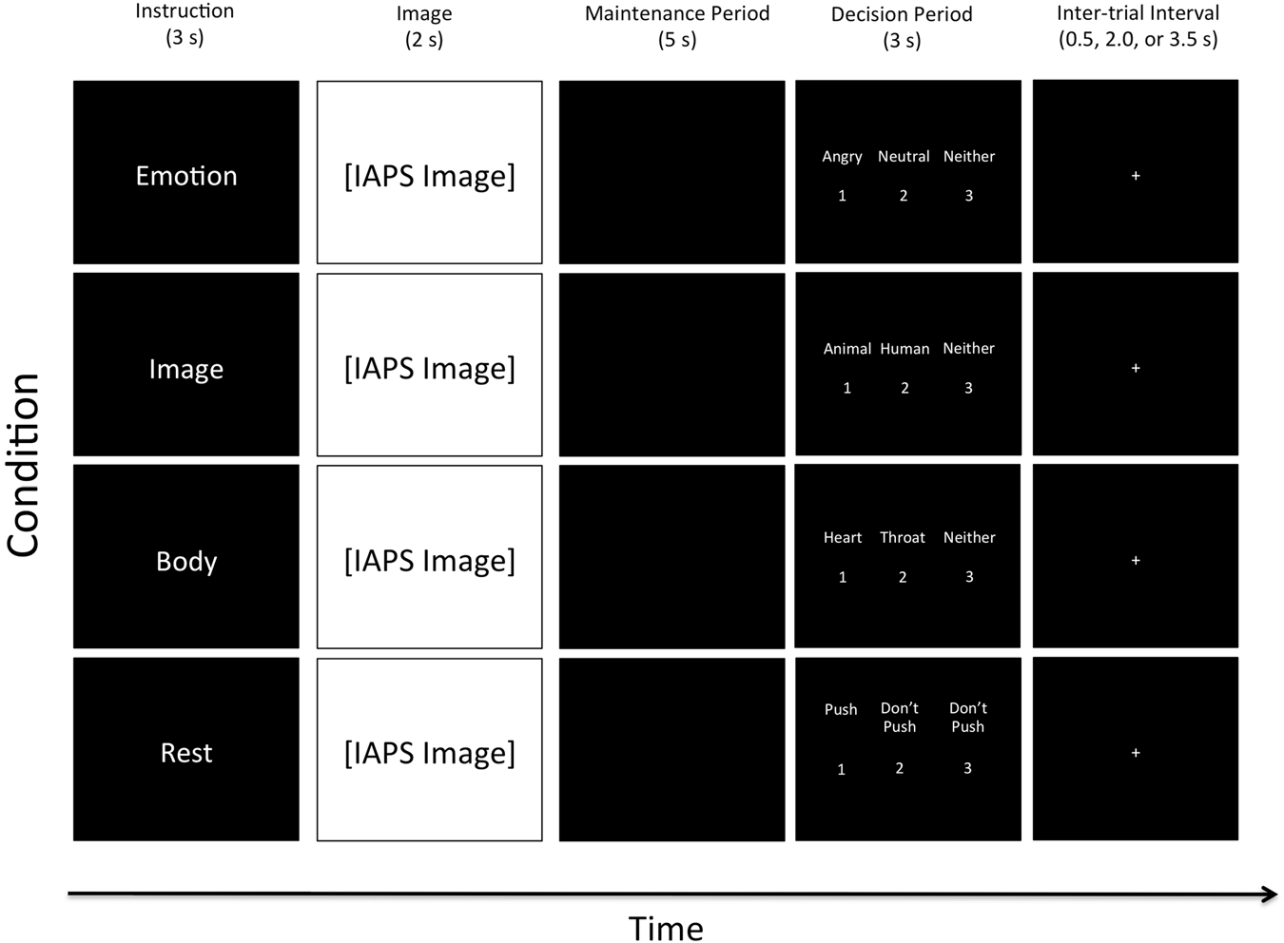
Illustration of the four task conditions. After the appearance of each instruction, an emotionally pleasant, unpleasant, or neutral image (from the International Affective Picture System [IAPS]) was presented followed by a maintenance period. All contrasts reported in this manuscript compare the 5-second maintenance periods between the “Emotion,” “Image,” and “Rest” conditions. Analyses of the “Body” condition will be presented in a separate manuscript. The decision period that followed included making a simple identification judgment from memory that included 3 options (where the correct answer was different depending on the instruction associated with that condition; described in greater detail in the text). Participants did not know what condition-specific options would be presented on a given trial, but could select “Neither” if the available options on that trial were both incorrect.

Upon completing the informed consent, participants were presented with written instructions (on a laptop computer) for how to perform the WM task (this task is illustrated in Fig. 1). These instructions stated “you will be shown a series of pictures that typically trigger emotional reactions” and “on each trial you will be shown one picture and given instructions to pay attention to something specific.” The instructions then informed the participants that there would be a pause after seeing the picture (where only a black screen was shown), during which they would be required to maintain the attended item in memory. Next, participants were told that, after the pause, three options would appear on the screen, and that they would be asked to press one of three corresponding buttons in order to test their memory.

Before each trial, an instruction appeared (in pseudo-random order) stating either “Emotion,” “Image,” “Body,” or “Rest.” They were told the “Emotion” instruction meant “you should pay attention to your own emotional reaction to the picture and hold this emotional feeling in mind” during the pause. Participants were informed that, when the three options appeared on the screen after the pause, two options would be emotion words (including: angry, disgusted, happy, neutral, afraid, or sad). The third option would be “neither.” They were instructed to choose the option (by button press) that corresponded best to the emotional response they were holding in memory. They were told the “Image” instruction meant “you should pay attention to the things in the image and hold the image in mind” during the pause. Participants were informed that, when the three options appeared on the screen after the pause, two options would be category words (including: human, animal, child, adult, male, female, living, or non-living only). The third option would be “neither.” They were instructed to choose the option (by button press) that corresponded best to their memory of what was in the image. They were told the “Body” instruction meant “you should pay attention to your own physical bodily reaction to the picture and hold this bodily feeling in mind” during the pause. Participants were informed that, when the three options appeared on the screen after the pause, two options would correspond to places on their body where they may have felt a change (including: heart, stomach, arms, face, throat, or no change). The third option would be “neither.” They were instructed to choose the option (by button press) that corresponded best to their memory of the most prominent region where they felt a change in their body. Finally, they were told the “Rest” instruction meant “you do not need to remember anything” during the pause. Participants were informed that, when the three options appeared on the screen after the pause, two options would say “don’t push” and the third option would be say “Push.” They were instructed to choose the option (by button press) that said “Push” on each trial. This condition acted as a control condition in which nothing was held in WM during the maintenance period, but where all stimulus conditions were identical.

Finally, they were instructed to use particular strategies during the pause period for each trial type. For the “Emotion” condition, they were asked to “hold the emotional feeling in mind in order to remember what emotion it was.” For the “Image” condition, they were asked to “hold the visual image of the picture in mind in order to remember what was in it.” For the “Body” condition, they were asked to “hold the bodily feeling in mind in order to remember where you felt your body react.” They were also told to “try your best to NOT simply hold a word in mind instead“ (such as repeating “animal, animal, animal,” or “sad, sad, sad,” or “stomach, stomach, stomach” in order to remember). This was done to avoid the use of an auditory WM strategy in each condition (e.g., so that participants were actually holding in mind an emotion concept in the “Emotion” condition, a visual image in the “Image” condition, etc.). After reading these instructions, participants were offered an opportunity to ask questions, and then they were allowed to practice the task for several trials on the laptop. This practice period gave two exposures to each instruction type. After this practice period, participants could again ask any clarifying questions if something was still not fully understood.

Participants were then taken to the magnetic resonance imaging (MRI) scanner at the University of Arizona where they underwent functional MRI scanning (see Neuroimaging Methods below) while completing the WM task. Before scanning began, they were also given a small number of practice trials to become accustomed to performing the task inside the scanner environment.

The task used normative emotional stimuli acquired from the International Affective Picture System (IAPS). On the basis of the IAPS normative data (both male/female) provided by Lang *et al*.^38^ using a 9-point rating scale, images for each valence were selected (unpleasant (U) = M_valence_ < 4.0, neutral (N) = 4.0 < M_valence_ < 6.0, pleasant (P) = M_valence_ > 6.0). The task was counterbalanced to the greatest extent possible with respect to all stimulus and condition variables. This included ensuring that each response option was shown a roughly equivalent number of times. It also included ensuring that each task condition had an equivalent number of pictures in each valence category (i.e., each of the four attention/memory conditions included the presentation of 10 unpleasant pictures, 5 pleasant pictures, and 5 neutral pictures in pseudo-random order), and that these pictures were matched for content across conditions to the greatest extent possible. A greater number of normatively unpleasant pictures were included because there are a greater number of unpleasant basic emotion categories (i.e., “sad,” “afraid,” “angry,” and “disgusted” vs. only “happy” and “neutral”). Two counterbalanced task versions were also created, by interchanging the pictures used between the “Emotion” and “Image” conditions and between the “Body” and “Rest” conditions. Each participant performed one of these equivalent task versions (i.e., half of participants got version 1 and half got version 2). Thus, any potential influence of the different pictures seen within each condition would be expected to cancel out within group analyses.

Task length (20 minutes) allowed for 20 trials within each of the 4 conditions. On each trial, the timing was as follows: Trial Instruction = 3 s, Image = 2 s, Maintenance Period = 5 s, Decision Period (displaying the three options) = 3 s. After the decision period, there was also a variable-length inter-trial interval (displaying a crosshair), which was jittered so as to last either 0.5 s, 2 s, or 3.5 s.

After completing scanning, participants were then escorted back to the lab, seated at a laptop, and asked to complete some additional measures.

### Secondary Measures

#### Emotional Awareness Measures

Two measures of EA were taken. First, participants completed an on-line version of the levels of emotional awareness scale (LEAS) (www.eleastest.net) that makes use of a validated automatic scoring program^39^. The LEAS includes the presentation of 2–4 sentence descriptions of 20 social situations that each involve 2 people. The situation descriptions are designed to elicit four emotion categories (sadness, happiness, anger, and fear) at 5 levels of complexity. One situation is presented on each electronically presented page, followed by two questions: “How would you feel?” and “How would the other person feel?” Separate response boxes are provided for typing in the answers to each question. Participants are asked to type their responses using as much or as little space as needed to answer. They are also told that they must use the word “feel” in their responses.

EA level scores are assigned based on the words participants write in their responses. The lowest possible score is given to non-feeling words (Level 0). Words related to physiological sensations (e.g., “tired”) are given a level 1 score, whereas level 2 scores instead reflect feeling-related actions (e.g., “punching”) or simple valence discriminations (e.g., “bad,” “good”) that have inherent avoidance- or approach-related content. Level 3 scores are given to single emotion concept terms (e.g., “happy,” “sad”). Level 4 scores are given when at least 2 words from level 3 are used in the same item (i.e., conveying greater emotional differentiation than either word alone). The self- and other-related responses are scored separately for each item as described above (i.e., with a value of 0–4). A “total” score is also given for each of the 20 LEAS items; this score represents the higher of the self- and other-related scores, unless a score of 4 is given for both. When this happens, a total score of 5 is given for the item, as long as the self- and other-related responses are capable of being differentiated (for more detail, see ref.^29^). (Note: The LEAS scores from this data set have previously been published in conjunction with other neuroimaging data^21,40,41^. Their relation to imaging data from this EWM task, however, is novel to the present manuscript).

As a second measure of EA, participants also completed the Frith-Happé-Animations Task (AT;^42^). This task was originally designed to measure theory of mind more generally; however, it has also recently been used in a few studies, in conjunction with the LEAS scoring system, to provide a complementary measure of EA that does not depend on language-based prompts or require participants to imagine detailed scenarios (e.g., see refs^43–45^). As used in this study, the AT consisted of 12 animations of simple moving shapes (i.e., 2 triangles) that were presented on a computer screen (each lasting 34–45 seconds). These 12 animation clips fell into 3 categories including 4 animations each: 1) a “thoughts/feelings” (TF) category, with animations that promoted the perception of beliefs, desires, and emotions within the triangles; 2) a “simple interactions” (SI) category, with animations that promoted the perception of simple goal-directed movement (e.g., one triangle “following” another); and 3) a “random movement” (RM) category, which included animations of the triangles drifting around the screen with no meaningful pattern. Before viewing the animations (presented in counterbalanced order across participants), the participants were informed regarding the three categories of animations, and shown one example animation of each type. They were then told to relax and watch each animation and to “describe what was happening in the animation” by typing a description into a textbox provided on the computer directly after viewing each clip. Half of the animations (two from each category) were preceded by a verbal cue informing them of the animation type (i.e., TF, SI, or RM), while the other half were not preceded by this information.

To evaluate EA, each of the written animation descriptions was coded and scored according to the criteria for scoring the LEAS (e.g., as also done in refs^43,44^), using the previously validated automatic LEAS scoring program^39^. A research assistant also subsequently examined the written descriptions and automatic scores, and corrected any false positives or false negatives in the output of the automated program (according to the LEAS scoring manual). However, as there was no “self” and “other” within the animations, a level 5 score for each written description was not provided. Each animation description therefore received an EA level score of 0–4, and these scores were then summed over the 12 animation descriptions for each individual. This second method of evaluating emotional awareness used stimulus prompts that were visual as opposed to the language-based prompts describing social scenarios used by the LEAS, and may therefore be less confounded by individual differences in linguistic- or imagination-related capacities.

### Neuroimaging Methods

A 3T Siemens Skyra scanner (Siemens, Erlangen, Germany), with a 32-channel head coil, was used to perform neuroimaging. T1-weighted structural 3D MPRAGE images were acquired (TR/TE/flip angle = 2.1 s/2.33 ms/12 degree) covering 176 sagittal slices (256 × 256) and had a slice thickness of 1 mm (voxel size = 1 × 1 × 1). Functional T2*-weighted scans were acquired over 32 transverse slices (2.5 mm thickness). Each volume was collected using an interleaved sequence (TR/TE/flip angle = 2.0 s/25 ms/90 degree). The voxel size of the T2* sequence was 2.5 × 2.5 × 3.5 mm. The field of view (FOV) was 240 mm.

### Image processing

Preprocessing steps, as well as subsequent statistical analyses, were performed using SPM12 (Wellcome Department of Cognitive Neurology, London, UK; http://www.fil.ion.ucl.ac.uk/spm) for all MRI scans. Using standard algorithms, raw functional images were realigned, unwarped, and coregistered to each subject’s MPRAGE image. The images were then normalized to Montreal Neurological Institute (MNI) coordinate space, spatially smoothed to 6 mm (full-width at half maximum), and resliced to 2 × 2 × 2 mm voxels. The standard canonical hemodynamic response function in SPM was used, and low-frequency confounds were minimized with a 128-second high-pass filter. Serial autocorrelation was further corrected using the AR(1) function. The Artifact Detection Tool (ART; http://www.nitrc.org/projects/artifact_detect/) was also used to regress out scans as nuisance covariates in the first-level analysis (threshold: 3 SD in mean global intensity and scan-to-scan motion that exceeded 1.0 mm).

### Statistical Analysis

For each participant, a general linear model was specified to contrast activation during the maintenance period between the “Emotion,” “Image,” and “Rest” conditions. Contrasts involving the “Body” condition will be reported in a separate manuscript (in preparation). Each trial was modeled as a 5-second interval. Motion regressors (generated by ART – see image processing above) were also added to each of these 1^st^-level designs. These contrast images were then entered into second-level SPM analyses (one-sample T-tests) to assess the main effect of each contrast of interest. The first contrast was “Emotion > Image,” which should highlight all regions activated by maintaining emotions that are not also activated by maintaining visual information. The second contrast was “Emotion > Rest,” which should highlight all regions activated by maintaining one’s own emotions (i.e., relative to a period involving no WM maintenance). The third contrast was “Image > Rest,” which should highlight all regions activated by maintaining the visual images (i.e., relative to no WM maintenance). The latter two contrasts, and their inverses, were analyzed in order to allow for more thorough interpretation of the primary “Emotion > Image” contrast, which itself replicates the contrasts done between emotion-focused and vision-focused attention (e.g. ref.^13^) and WM^20^ in previous studies. Finally, conjunction analyses were performed (within a Flexible Factorial model in SPM12) to confirm regions of activation common to (1) the “Emotion > Rest” and “Image > Rest” contrasts, and (2) the “Rest > Emotion” and “Rest > Image” contrasts. These conjunction analyses were performed using SPM12’s “conjunction null” function^46^.

For these analyses we set a whole-brain peak significance threshold of p < 0.001 (uncorrected), and a cluster extent threshold of *p* < 0.05 (false discovery rate [FDR] corrected). The first eigenvariate across subjects was also extracted from the dACC cluster found in the “Emotion > Image” contrast (using SPM12’s built-in volume-of-interest [VOI] time-series extraction tool; see results section) that was closest to the region observed in previous EA studies^32,33^, and this was correlated with our two EA measures (described further below). Cluster identification/labeling was done in conjunction with the Automated Anatomical Labeling (AAL) atlas within SPM12^47^.

## Results

### fMRI Activation Contrasts

#### Maintenance Period: Emotion > Image

**Figure 2:**
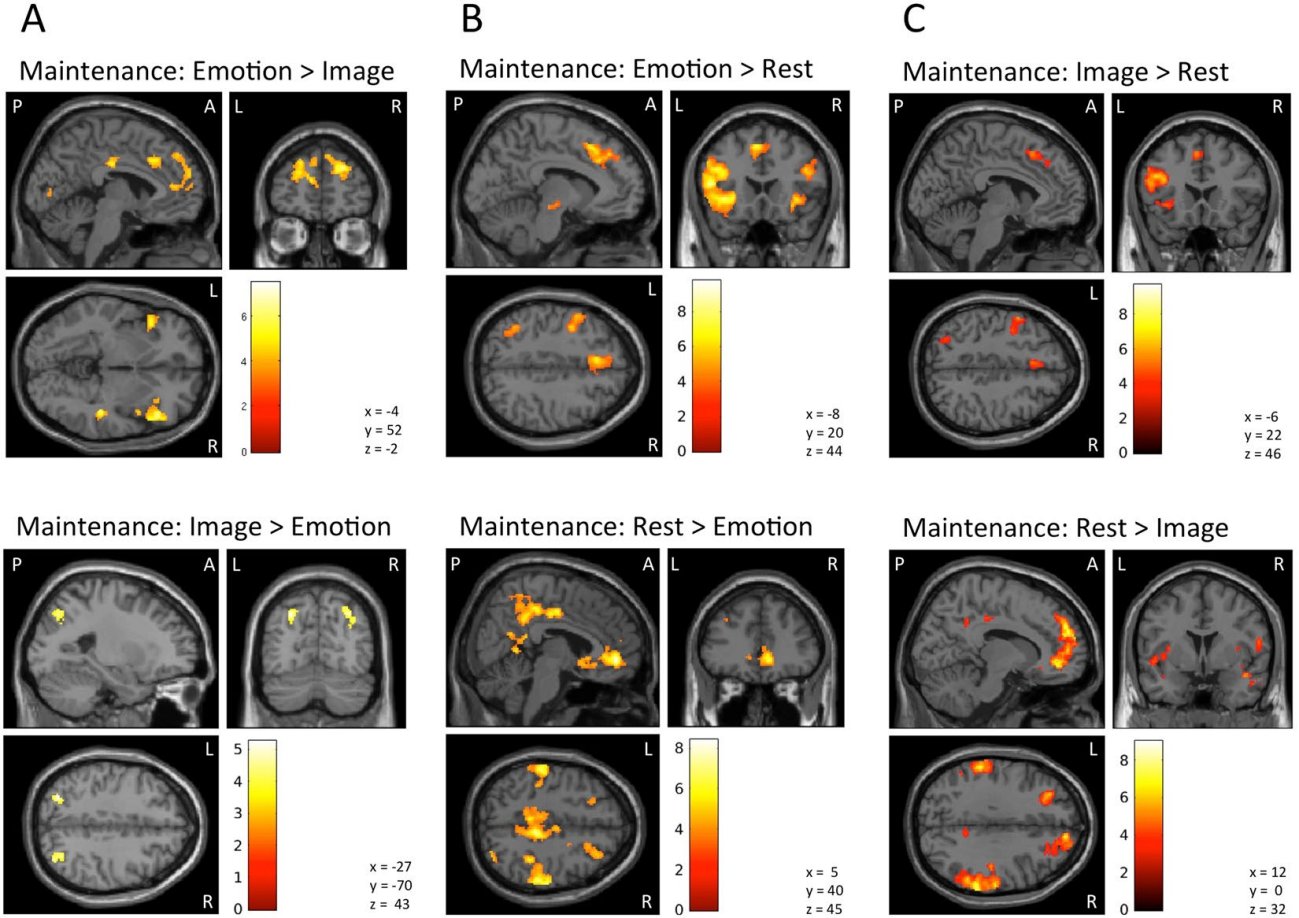
Illustration of the imaging results contrasting the maintenance period of the (**A**) “Emotion” and “Image” conditions, (**B**) “Emotion” and “Rest” conditions, and (**C**) the “Image” and “Rest” conditions. Images are thresholded using a peak threshold of p < 0.001 (uncorrected) and a cluster threshold of p < 0.05, FDR-corrected. Images are shown in neurological orientation (L = left; R = right; P = Posterior; A = Anterior).

This contrast revealed 11 clusters, spanning the left anterior insula and ventrolateral prefrontal cortex (AI/VLPFC), right AI/VLPFC (2 clusters), dACC (bilaterally), left posterior parietal cortex (PPC), mid-/posterior cingulate cortex, right dorsal MPFC (DMPFC), left DMPFC and rostral ACC (rACC), right PPC, right posterior temporal cortex, and a bilateral region of the primary visual cortex (for AAL atlas labels, see Table 1; Fig. 2A).

**Table 1:** fMRI Results: Emotion vs. Image.

Brain Region	AAL Atlas Labels	Peak Voxel Coordinate	Cluster Size (k_E_)	T-score
***Emotion *>* Image**** (FDR-corrected cluster threshold*, *p* < *0.05)*				
Left AI/VLPFC	Frontal_Inf_Orb_2_L OFCpost_L Temporal_Pole_Sup_L Frontal_Inf_Tri_L Insula_L Frontal_Inf_Oper_L	−34, 18, −20	614	7.47
Right AI/VLPFC	Frontal_Inf_Orb_2_R OFCpost _R Frontal_Inf_Tri_R Temporal_Pole_Sup_R Frontal_Inf_Oper_R Insula_R	50, 26, −2	446	7.40
Mid-/Posterior Cingulate Cortex (Bilateral)	Cingulate_Mid_L Cingulate_Mid_R	2, −20, 32	147	6.72
Right DMPFC	Frontal_Mid_2_R Frontal_Sup_Medial_R Frontal_Sup_2_R	24, 54, 30	432	6.71
Right Posterior Temporal Cortex	Temporal_Sup_R Temporal_Mid_R	46, −30, −2	216	6.47
Left DMPFC/rACC	Frontal_Mid_2_L Cingulate_Ant_L Frontal_Sup_Medial_L Frontal_Sup_2_L	−10, 54, 18	830	5.90
dACC (Bilateral)	Cingulate_Ant_L Cingulate_Mid_R Cingulate_Mid_L Frontal_Sup_Medial_L Frontal_Sup_2_L	−6, 20, 38	206	5.85
Right PPC	Angular_R SupraMarginal_R Parietal_Inf_R	58, −58, 32	90	5.53
Right AI	Frontal_Inf_Tri_R Frontal_Inf_Orb_2_R Insula_R	34, 32, 4	72	5.34
Primary Visual Cortex (Bilateral)	Lingual_L Calcarine_R Calcarine_L	0, −82, 4	66	5.10
Left PPC	Parietal_Inf_L Angular_L	−48, −60, 50	120	4.80
***Image *>* Emotion**** (FDR-corrected cluster threshold, p* < *0.05)*				
Left Occipital-Parietal Cortex	Parietal_Inf_L Parietal_Sup_L Occipital_Mid_L Occipital_Sup_L	−26, −70, 44	96	5.26
Right Occipital-Parietal Cortex	Parietal_Sup_R Occipital_Mid_R Occipital_Sup_R Angular_R	34, −74, 44	215	5.16

The reverse contrast (Image > Emotion) instead highlighted clusters spanning the left and right occipital-parietal cortex (for AAL atlas labels, see Table 1; Fig. 2A).

#### Maintenance Period: Emotion > Rest

This contrast revealed 7 clusters, which spanned the left AI/VLPFC, and the dorsolateral PFC (DLPFC), the right AI, the posterior dorsomedial frontal cortex and supplementary motor area (DMFC/SMA; bilaterally), the left posterior temporal cortex, the left midbrain, the right DLPFC, and the left PPC (for AAL atlas labels, see Table 2; Fig. 2B).

**Table 2:** fMRI Results: Emotion vs. Rest.

Brain Region	AAL Atlas Label	Peak Voxel Coordinate	Cluster Size (k_E_)	T-score
***Emotion *>* Rest**** (FDR-corrected cluster threshold, p *<* 0.05)*				
Left AI/VLPFC/DLPFC	Frontal_Inf_Oper_L Insula_L Frontal_Inf_Orb_2_L Frontal_Inf_Tri_L Precentral_L OFCpost_L Temporal_Pole_Sup_L Frontal_Mid_2_L OFClat_L	−50, 16, 12	3867	9.76
Right AI	Frontal_Inf_Tri_R Insula_R Frontal_Inf_Orb_2_L	32, 26, −2	231	7.14
Posterior DMFC/SMA (Bilateral)	Frontal_Sup_Medial_R Cingulate_Mid_R Supp_Motor_Area_L Supp_Motor_Area_R Frontal_Sup_Medial_L Cingulate_Ant_L Cingulate_Mid_L Frontal_Sup_2_L	−4, 20, 48	730	7.12
Left Posterior Temporal Cortex	Temporal_Mid_L	−48, −44, 0	244	6.94
Left Midbrain (Substantia Nigra)	No AAL Atlas Label	−10, −22, −12	69	6.26
Right DLPFC	Frontal_Inf_Tri_R Frontal_Inf_Oper_R Frontal_Mid_2_R	46, 22, 26	226	5.86
Left PPC	Parietal_Inf_L Parietal_Sup_L Angular_L Occipital_Mid_L	−38, −54, 44	241	5.24
***Rest *>* Emotion**** (FDR-corrected cluster threshold, p *<* 0.05)*				
VMPFC/rACC/sgACC (Bilateral)	Frontal_Med_Orb_R Frontal_Sup_Medial_R Frontal_Sup_2_R Cingulate_Ant_R Caudate_R Olfactory_R Frontal_Med_Orb_L Frontal_Sup_Medial_L Cingulate_Ant_L Olfactory_L	8, 48, −6	1025	8.4
Right Lateral Parietal Cortex	Angular_R Temporal_Pole_Mid_R Parietal_Sup_R Temporal_Pole_Sup_R Occipital_Inf_R SupraMarginal_R Parietal_Inf_R Postcentral_R Temporal_Sup_R Temporal_Mid_R Temporal_Inf_R Rolandic_Oper_R Occipital_Sup_R Occipital_Mid_R	60, −36, 38	4059	7.96
Posterior Cingulate Cortex/Precuneus	Cingulate_Mid_R Cingulate_Post_R Paracentral_Lobule_L Supp_Motor_Area_R Paracentral_Lobule_R Parietal_Sup_R Precuneus_R Cingulate_Mid_L Precuneus_L	8, −32, 46	1729	7.29
Left Posterior Insula	Insula_L Temporal_Sup_L Heschl_L Rolandic_Oper_L	−38, −16, 0	484	7.22
Left Lateral Parietal Cortex	Parietal_Inf_L SupraMarginal_L Temporal_Sup_L Postcentral_L Rolandic_Oper_L	−58, −24, 44	1153	6.89
Left Parahippocampal Cortex	ParaHippocampal_L Fusiform_L Temporal_inf_L Hippocampus_L	−30, −36, −14	97	6.82
Right Retrosplenial Cortex	Precuneus_R Vermis_4_5 Cuneus_R Cingulate_Post_R Calcarine_R Lingual_R	18, −54, 20	284	6.09
Right Superior Frontal Sulcus	Frontal_Sup_2_R Frontal_Mid_2_R Precentral_R	22, 20, 44	288	5.79
Right Hippocampus	Hippocampus_R Parahippocampal_R	30, −26, −12	52	5.75
Left Occipital-Parietal Cortex	Occipital_Mid_L Angular_L Parietal_Inf_L Occipital_Sup_L	−40, −76, 40	220	5.66
Left Superior Frontal Sulcus	Frontal_Sup_2_L Frontal_Mid_2_L	−24, 22, 40	176	5.13
Right Parahippocampal Cortex	ParaHippocampal_R Fusiform_R Hippocampus_R	30, −40, −10	61	5.11
Right Posterior Insula	Insula_R Putamen_R Temporal_Sup_R	38, −16, 0	126	4.99
Left Retrosplenial Cortex	Cuneus_L Precuneus_L Calcarine_L	−18, −58, 20	62	4.96
Right Fusiform Gyrus	Fusiform_R Cerebelum_4_5_R Cerebelum_6_R	24, −50, −18	45	4.95
Right Ventral Putamen	Putamen_R Rectus_5	22, 16, −6	74	4.88
Right Frontal Operculum	Rolandic_Oper_R Frontal_Inf_Oper_R Precentral_R	54, 2, 12	76	4.84
Right Superior Insula	Insula_R Putamen_R Rolandic_Oper_R	36, 6, 6	45	4.83
Left Lateral Occipital Cortex	Occipital_Mid_L Temporal_Mid_L	−50, −66, 2	49	4.78
Left Lateral Occipital Cortex	Occipital_Mid_L	−42, −76, 4	53	4.63
Left Central Sulcus	Postcentral_L Parietal_Sup _L	−34, −40, 58	52	4.53

The reverse contrast (Rest > Emotion) instead highlighted several clusters spanning the bilateral ventromedial PFC (VMPFC), rACC, subgenual ACC (sgACC), right and left lateral parietal regions, right and left posterior cingulate cortex and precuneus, right and left posterior insula, among other regions (see Table 2; Fig. 2B). This overall pattern of activation overlaps considerably with the regions that make up the DMN, and which are known to be more active during the resting state and other states not involving a goal-directed task^48^.

#### Maintenance Period: Image > Rest

This contrast revealed 3 clusters, which spanned regions of the left DLPFC/VLPFC/AI, the left posterior DMFC/SMA, and the left occipital-parietal cortex (for AAL atlas labels, see Table 3; Fig. 2C).

**Table 3:** fMRI Results: Image vs. Rest.

Brain Region	AAL Atlas Label	Peak Voxel Coordinate	Cluster Size (k_E_)	T-score
***Image *>* Rest**** (FDR-corrected cluster threshold, p* < *0.05)*				
Left DLPFC/VLPFC/AI	Frontal_Inf_Tri_L Precentral_L Frontal_Inf_Orb_2_L Frontal_Mid_2_L OFCpost_L Insula_L Frontal_Inf_Oper_L	−40, 14, 28	2090	9.69
Left Posterior DMFC/SMA	Supp_Motor_Area_L Frontal_Sup_Medial_L	−4, 18, 50	138	5.33
Left Occipital-Parietal Cortex	Parietal_Sup_L Parietal_Inf_L Occipital_Mid_L	−30, −68, 50	116	4.79
***Rest *>* Image**** (FDR-corrected cluster threshold, p *<* 0.05)*				
VMPFC/DMPFC/dACC/rACC/sgACC (Bilateral)	Frontal_Med_Orb_R Frontal_Mid_2_R Caudate_R Frontal_Sup_2_L Frontal_Sup_Medial_R Cingulate_Ant_R Olfactory_L Olfactory_R Frontal_Sup_2_R Frontal_Med_Orb_L Frontal_Sup_Medial_L Cingulate_Ant_L	10, 46, −6	2960	9.02
Right Lateral Parietal Cortex/Right Posterior Insula	SupraMarginal_R Rolandic_Oper_R Insula_R Angular_R Temporal_Pole_Mid_R Temporal_Pole_Sup_R Precentral_R Parietal_Inf_R Postcentral_R Putamen_R OFCpost_R Temporal_Mid_R Temporal_Inf_R Frontal_Inf_Oper_R Heschl_R Temporal_Sup_R	66, −38, 28	3900	7.99
Left Lateral Parietal Cortex	Parietal_Inf_L Temporal_Sup_L SupraMarginal_L Angular_L Rolandic_Oper_L Postcentral_L	−62, −40, 40	917	7.59
Right VLPFC	Frontal_Inf_Tri_R Frontal_Mid_2_R Frontal_Inf_Orb_2_R	48, 40, 4	374	7.57
Left Posterior Insula	Insula_L Temporal_Sup_L Temporal_Mid_L Temporal_Pole_Sup_L Heschl_L Rolandic_Oper_L	−42, −16, 0	574	7.18
Left Posterior Cingulate Cortex	Cingulum_Mid_L	−10, −26, 40	184	6.51
Left Superior Frontal Sulcus	Frontal_Mid_2_L Frontal_Sup_2_L	−30, 36, 32	301	6.06
Right Posterior Cingulate Cortex	Cingulate_Mid_ Cingulate_Mid_L Cingulate_Post_R Precuneus_L	14, −28, 40	208	4.8

The reverse contrast (Rest > Image) instead highlighted many of the same DMN regions found for the “Rest > Emotion” contrast (see Table 3; Fig. 2C). However, this contrast also activated DMPFC and dACC regions not observed in the “Rest > Emotion” contrast.

#### Conjunction analyses

The first conjunction analysis revealed 6 clusters common to the “Emotion > Rest” and “Image > Rest” contrasts (See Table 4). These clusters spanned a set of regions commonly activated by WM tasks, including the DLPFC, DMFC/SMA, and AI bilaterally, as well as the left VLPFC and left occipital-parietal cortex. To provide additional confirmation that these voxel clusters in fact overlapped with those commonly observed across previous WM studies, we also performed an automated, term-based meta-analysis of previous working memory studies – using the “Neurosynth” software package (http://neurosynth.org)^49^ – and compared these voxel-wise meta-analytic results to those reported in Table 4. The search term “working memory” yielded 901 relevant neuroimaging studies of WM within the NeuroSynth database (conducted on 1/31/18), and the subsequent meta-analysis revealed a forward inference map containing voxel cluster within all of the brain regions reported in the conjunction analysis described above. Specifically, an inclusive masking analysis within SPM12 revealed that 85.4% of the significant voxels observed in our conjunction analysis (i.e., 2996 out of the 3508 voxels reported in Table 4) overlapped with significant voxels in the forward inference map calculated within Neurosynth. A substantial percentage of overlapping voxels was also present for each of the 6 reported clusters: Left DLPFC/VLPFC (83.0%); DMFC/SMA (80.8%); Left Occipital-Parietal Cortex (98.8%); Left AI (97.1%); Right DLPFC (81.8%); Right AI (100%).

**Table 4:** Conjunction Analyses.

Brain Region	AAL Atlas Label	Peak Voxel Coordinate	Cluster Size (kE)	T-score
**Activation clusters common to the Emotion > Rest and Image > Rest contrasts** (FDR-corrected cluster threshold, p < 0.05)				
Left DLPFC/VLPFC	Frontal_Mid_2_L Frontal_Inf_Tri_L Frontal_Inf_Oper_L Precentral_L	−40, 14, 28	2207	10.59
Posterior DMFC/SMA (Bilateral)	Frontal_Sup_Medial_R Supp_Motor_Area_R Cingulate_Mid_R Frontal_Sup_Medial_L Supp_Motor_Area_L	−4, 20, 48	449	7.17
Left Occipital-Parietal Cortex	Angular_L Parietal_Inf_L Parietal_Sup_L Occipital_Mid_L	−36, −56, 46	333	5.40
Left AI	Frontal_Inf_Orb_2_L Insula_L Frontal_Inf_Tri_L	−32, 24, 0	242	5.32
Right DLPFC	Frontal_Inf_Tri_R Frontal_Inf_Oper_R Frontal_Mid_2_R	46, 26, 26	214	4.89
Right AI	Frontal_Inf_Orb_2_R Insula_R	30, 24, 0	63	4.81
**Activation clusters common to the Rest > Emotion and Rest > Image contrasts** (FDR-corrected cluster threshold, p < 0.05)				
VMPFC/rACC/sgACC (Bilateral)	Cingulum_Ant_L Cingulum_Ant_R Frontal_Med_Orb_R Frontal_Med_Orb_L Frontal_Sup_2_L Frontal_Sup_Medial_R Olfactory L Frontal_Sup_Medial_L	6, 48, −4	1074	7.89
Left Posterior Cingulate	Paracentral_Lobule_L Cingulate_Mid_L	−10, −26, 40	248	7.81
Right Lateral Parietal Cortex	Angular_R Parietal_Inf_R Temporal_Mid_R Rolandic_Oper_R Temporal_Sup_R Postcentral_R Supramarginal_R	58, −36, 40	1812	6.78
Left Lateral Parietal Cortex	Temporal_Sup_L SupraMarginal_L Parietal_Inf_L Rolandic_Oper_L Postcentral_L	−58, −34, 34	844	6.58
Right Middle Temporal Gyrus	Temporal_Mid_R Temporal_Sup_R	62, −16, −8	111	5.71
Left Posterior Insula	Temporal_Pole_Sup_L Heschl_L Insula_L Rolandic_Oper_L Temporal_Sup_L Temporal_Mid_L	−40, −16, 0	424	5.67
Left Superior Frontal Sulcus	Frontal_Mid_2_L Frontal_Sup_2_L	−30, 34, 34	139	5.63
Right Posterior Cingulate Cortex	Cingulate_Post_R Paracentral_Lobule_R Cingulum_Mid_R Precuneus_L Precuneus_R	14, −28, 40	363	5.46
Right Motor Cortex	Heschl_R Frontal_Inf_Oper_R Rolandic_Oper_R Temporal_Pole_Sup_R Precentral_R	58, 8, 16	123	5.06
Right Posterior Insula	Putamen_R Rolandic_Oper_R Insula_R	36, 6, 8	68	4.94
Right Posterior Temporal Cortex	Temporal_Inf_R Temporal_Mid_R	56, −58, 0	213	4.78
Right Posterior Insula	Putamen_R Insula_R Temporal_Pole_Sup_R Temporal_Sup_R	40, −6, −8	126	4.77
Right VLPFC	Frontal_Inf_Tri_R Frontal_Mid_2_R	42, 42, 10	61	4.70
Right Inferior Temporal Gyrus	Temporal_Inf_R Temporal_Mid_R	52, −8, −26	56	4.67

The second conjunction analysis revealed 14 clusters common to the “Rest > Emotion” and “Rest > Image” contrasts (See Table 4). These included a set of DMN regions that largely overlapped with those reported in the “Rest > Emotion” contrast above, and included the VMPFC (but not the DMPFC/dACC).

### Cognitive/Behavioral Measures

The “Image” condition of the WM task had an average response accuracy of 92.0% (SD = 7.3%). The “Rest” condition had an average response accuracy of 99.0% (SD = 1.8%). As there currently exists no means of objectively measuring the basic emotion category or bodily reaction that was actually experienced, we were not able to assess accuracy within the “Emotion” and “Body” conditions.

LEAS total scores had a mean of 73.7 (SD = 9.68). AT total scores had a mean of 11.63 (SD = 5.5). There was a significant positive correlation between LEAS total scores and AT total scores (r = 0.582, p = 0.001).

**Figure 3:**
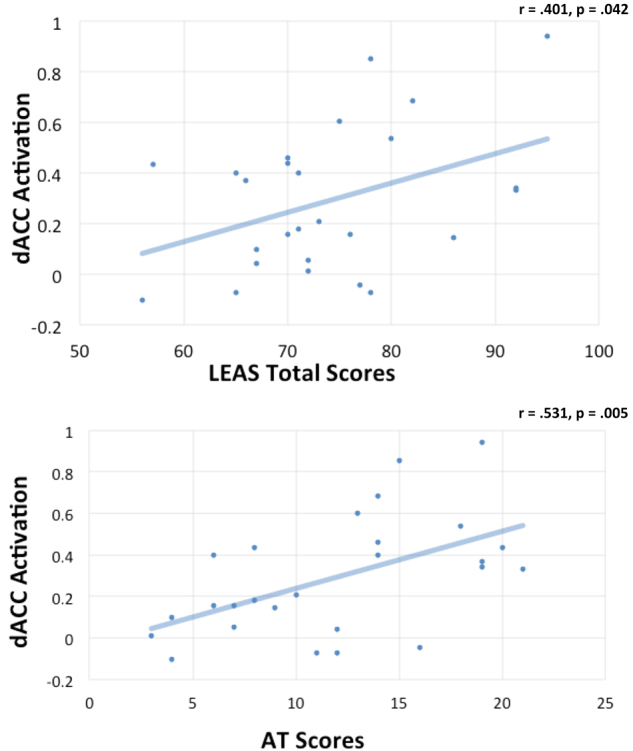
Scatterplots illustrating the significant positive relationships observed between participants’ dorsal anterior cingulate cortex (dACC) activation in the “Emotion > Image” contrast and two measures of their emotional awareness. The top panel illustrates this relationship with Levels of Emotional Awareness Scale (LEAS) Total scores. The bottom panel illustrates this relationship with scores on the “animations task” (AT) described in the main text.

### Correlations between EA measures and dACC activation

In line with our hypothesis, dACC activation during the “Emotion > Image” contrast (i.e., the first eigenvariate extracted from the whole “dACC (bilateral)” cluster in Table 1; peak voxel = −6, 20, 38) was significantly positively correlated with LEAS total scores (r = 0.401, p = 0.042) and AT total scores (r = 0.531, p = 0.005)(See Fig. 3).

## Discussion

### Emotional Working Memory vs. Visual Working Memory

In this study, we extended a widely used emotion-focused attention task^13^ to include a WM delay period. When contrasting the WM maintenance period between the “Emotion” and “Image” conditions, we were able to replicate the previous results found in contrasts of emotion-focused and vision-focused attention. Specifically, as in the original study contrasting attentional conditions^13^, the “Emotion > Image” contrast here also revealed DMPFC regions (including both dACC and rACC); the reverse contrast also replicated the occipital-parietal clusters observed in that study, which are thought to play an important role in visuospatial attention^50^. This supports the idea that, as with the visual/auditory domain, there is also considerable overlap between WM- and attention-related neural mechanisms in the emotional domain. The role of DMPFC regions in reflecting on emotions was also further supported by our replication of previous findings that EA, as measured by both the LEAS and the AT, was positively correlated with the dACC activation revealed by this contrast^32,33^.

It is noteworthy that MPFC involvement was found here even though our task asked participants to focus on emotion concepts. This suggests that the increases vs. decreases in MPFC involvement observed in the previous studies of emotional WM (discussed in the introduction) are unlikely to be explained by the differential focus on emotion concepts (in ref.^20^) vs. emotional intensity (in ref.^22^). Therefore, our alternative hypothesis – that MPFC activation in WM depends on internal/self-related vs. external/other-related information – appears to gain greater support (for a similar proposal regarding the neural basis of internal vs. external information processing, see ref.^31^). This is also consistent with previous work linking MPFC to other internally focused/self-related cognitive processes^27^. Thus, the hypothesis that MPFC activation represents internal/self-related information, and therefore plays an important role in self-focused EWM (i.e., but not in other-focused EWM), appears supported.

### Emotional and Visual Working Memory vs. Passive Viewing

To further understand our results, however, we also made use of a baseline “Rest” condition requiring no top-down attentional or WM involvement (i.e., directed at either vision or emotion). When we contrasted this “Rest” condition with the “Emotion” and “Image” conditions, a more nuanced pattern of results emerged. First, both the “Emotion > Rest” and the “Image > Rest” contrasts revealed considerable ECN region involvement (i.e., as also supported by the large percentage of significant voxels we observed in the conjunction analysis of these two contrasts that overlapped with those of a meta-analysis of previous neuroimaging studies of WM). This confirms that ECN regions known to be involved in visual/auditory WM are also strongly activated by emotional WM (i.e., as also found in refs^20–22^). Second, both the “Rest > Emotion” and “Rest > Image” contrasts revealed VMPFC and other regions known to be involved in the DMN. Thus, these DMN regions (i.e., those other than DMPFC) appear to be inhibited regardless of whether one is maintaining visual or emotional information (i.e., as in ref.^20^). Finally, DMPFC regions were highlighted by the “Rest > Image” contrast, but *not* by the “Rest > Emotion” contrast; these regions were also not highlighted in the conjunction analysis of these two contrasts. When combined with our initial results of the “Emotion > Image” contrast, this overall pattern of results entails that: (1) DMPFC has a relatively high level of activity in the “Rest” condition (i.e., in which participants are exposed to emotional images but need not attend to or maintain any information), (2) DMPFC has a significantly lower level of activity during the “Image” condition (when presumably its role in representing automatic emotional responses to the images is suppressed), and (3) DMPFC activity *remains high during the “Emotion” condition*, which is why it is highlighted within the “Emotion > Image” contrast.

When considering these results together with those from our previous study of WM for the emotions perceived in others (i.e., other-focused EWM^21^), we suggest that WM for one’s own emotions (i.e., self-focused EWM) is best understood to involve both ECN and DMPFC regions (i.e., presumably, the ECN is maintaining self-related emotion representations in DMPFC). In contrast, other-focused EWM may not require DMPFC activation (but still involves ECN activation), perhaps because other-focused EWM still draws mainly from external perceptual (as opposed to internal/self-focused) information sources (i.e., as previously suggested in ref.^21^; it is also worth noting in this context that DMPFC has been implicated in the goal-directed retrieval of internally represented information more generally^31^). As both types of emotional and visual WM also involve reductions of activation in ventral MPFC regions of the DMN, this appears to further resolve the apparent discrepancy between the previous emotional WM studies discussed above^20,22^. This is because Waugh *et al*.^22^ highlighted more dorsal MPFC activation, whereas the study by Xin and Lei^20^ instead found reductions in a relatively larger MPFC cluster that also included the ventral regions, which our results suggest are inhibited during EWM. These findings are therefore both consistent with our pattern of results. Together, therefore, all of these findings can be accounted for if (1) all types of WM activate ECN regions and inhibit VMPFC (and other DMN regions), and (2) self-focused EWM also requires the activation of DMPFC (including dACC/rACC; i.e., due to the internally focused, self-related nature of this subtype of EWM), whereas visual WM and other-focused EWM does not.

### Limitations and Conclusions

Despite offering these potentially clarifying results, the present study also has some limitations that are important to consider. First, we could not assess accuracy levels within the “Emotion” condition, because there is no known objective and independent means of measuring what category of emotional response participants truly experienced (the general idea of “correct” emotional responses in our task is also questionable, given that previous studies have found that the IAPS stimuli we used lead to the self-report of a wide variety of different discrete emotions in different individuals; e.g. ref.^51^). However, the very high levels of accuracy within the “Image” and “Rest” conditions suggest that participants remained engaged and performed the task as instructed. In addition, the idea that the “Emotion” condition of the task was performed appropriately is further supported by the fact that we observed the same pattern of DMPFC activation found in previous studies of attention to one’s own emotions (e.g. refs^13,17^), as well as by the fact that DMPFC activity (along with that of other DMN regions) has previously been linked to emotion conceptualization processes (e.g. refs^12,23,52^).

Second, for the sake of simplicity, in the analyses presented we chose to collapse across the different valence/emotion categories of the images/responses, and to focus mainly on broader emotional vs. non-emotional content domains. As a result, we cannot rule out that these factors may have influenced our results. However, the images were matched for content/valence across the conditions (i.e., there was an equal number of normatively pleasant, unpleasant, and neutral pictures in each), and image-condition pairings were counterbalanced across participants, so it is likely that any effects on neural activation would have canceled out in the between-condition contrasts we presented. We have also previously shown that, even in the case of judging one’s own emotions as “neutral,” attention to emotion activates MPFC (i.e., relative to visual attention;^17^); thus, the inclusion of normatively neutral images in the “Emotion” condition would not be expected to influence MPFC involvement in a manner that would affect our interpretations/conclusions.

Third, it is important to highlight that our study design did not allow us to gather subjective ratings of the intensity of the affective responses triggered by viewing the emotion-provoking images (e.g., asking participants to provide intensity ratings at the end of each task trial would have considerably altered, and added to, the working memory demands of our task). Thus, while the images were matched for normatively rated emotional content across conditions/participants, we cannot rule out that emotional response intensity differed as a result of differing attentional focus in the “Emotion,” “Image,” and “Rest” conditions (although, as different studies have found variable effects of focusing on vs. away from emotion on response intensity, including increases, decreases, and no change^53–56^, there does not appear to be a strong basis for a specific a priori hypothesis about the expected direction of influence in these different attentional conditions). Future studies will therefore be necessary to confirm that the condition-specific neural activations we observed are not influenced by differences in subjective emotional intensity (i.e., as a result of differences in attentional focus).

Fourth, it should be mentioned that the strategy of the present study was to examine self-focused EWM as a means of building off of our previous study of other-focused EWM^21^. While considering the results of these two studies together has allowed us to suggest possible differences in MPFC involvement within these two types of EWM, future studies should more directly contrast self-focused and other-focused EWM in a single experimental design. This would represent an important next step toward increasing our understanding within this relatively new and under-investigated area of study. It will also be important to design paradigms that allow WM *manipulation* of self-related emotional information (i.e., as was done for other-focused EWM in our previous study^21^), as opposed to simply WM *maintenance* (as in this study), so that any potential differences between the neural basis of maintenance and manipulation of self-related emotional information can be examined.

One final limitation of the present study pertains to the unexpected (and somewhat unclear) result that the “Emotion > Rest” contrast did not reveal DMPFC activation. In retrospect, however, it is not surprising that DMPFC activity was relatively high in the “Rest” condition (i.e., leading to a non-significant difference between the “Emotion” and “Rest” conditions), given that previous work has shown that this region, and other DMN regions, typically show greater activation during resting conditions (i.e., as part of a “default” internal focus; e.g., see refs^30,31,48^). While we have suggested that the larger pattern of results we observed is consistent with the idea that self-focused EWM involves the goal-directed maintenance of DMPFC representations (i.e., even if such representations also remain somewhat activated due to an automatic internal focus at rest), it is also possible that our pattern of results could be interpreted in other ways. For example, one might think the DMPFC activation we observed could also be attributed to its known role in emotion perception/experience^57,58^. However, given that the contrasts discussed above revealed DMPFC activity during the delay period (where participants simply saw a black screen), it appears less plausible to attribute our results to current perception. Further, given that WM can be understood as the goal-directed maintenance of some of the same neural representations that contribute to perception/experience (i.e., as reviewed in the introduction section), there is no inconsistency in the idea that the same DMPFC representations are active during the perception, experience, and WM maintenance of emotional states. Nonetheless, it will be important for future research to examine this further, perhaps by testing the hypothesis that DMPFC activation increases with increasing self-focused EWM load (e.g., similar to the parametric increases in DMPFC activity previously observed with increasing “social working memory” load – another process that draws on internally stored information about the personality traits of others^59,60^).

In conclusion, this study found evidence that DMPFC regions (including rACC and dACC) may play an important role in the goal-directed maintenance of concept-level information about one’s own emotional responses in WM – and that this function may be linked to trait levels of EA. It also found evidence that ECN regions are engaged by, and VMPFC (and other DMN) regions are inhibited by, both vision-focused and emotion-focused WM. These results clarify the roles of these different regions/networks in emotion-focused WM. Given the potential role that this ability to voluntarily maintain and reflect upon one’s own emotions may play within emotional disorders and their treatments (e.g., in the context of psychotherapy;^23,36,37^), future studies should extend this paradigm to the investigation of psychiatric populations.

### Ethical approval

All procedures performed in studies involving human participants were in accordance with the ethical standards of the institutional and/or national research committee and with the 1964 Helsinki declaration and its later amendments or comparable ethical standards.

### Data availability

The datasets generated during and/or analyzed during the current study are available from the corresponding author upon reasonable request.
